# Deriving health state utilities for the numerical pain rating scale

**DOI:** 10.1186/1477-7525-9-96

**Published:** 2011-11-03

**Authors:** Simon Dixon, Chris D Poole, Isaac Odeyemi, Peny Retsa, Colette Chambers, Craig J Currie

**Affiliations:** 1 School of Health and Related Research (ScHARR), University of Sheffield, Sheffield, UK; 2 Global Epidemiology, Pharmatelligence, Cardiff, UK; 3 Health Economics and Outcomes Research, Astellas Pharma Europe Ltd, Staines, UK; 4 Department of Medicine, School of Medicine, Cardiff University, Cardiff, UK

**Keywords:** health economics, pain measurement, cost-effectiveness, quality of life

## Abstract

**Background:**

The use of patient reported outcome measures within cost-effectiveness analysis has become commonplace. However, specific measures are required that produce values, referred to as 'utilities', that are capable of generating quality adjusted life years. One such measure - the EQ-5D - has come under criticism due to the inherent limitations of its three-level response scales. In evaluations of chronic pain, the numerical pain rating scale (NPRS) which has eleven levels is routinely used which has a greater measurement range, but which can not be used in cost-effetiveness analyses. This study derived utility values for a series of EQ-5D health states that replace the pain dimensions with the NPRS, thereby allowing a potentially greater range of pain intensities to be captured and included in economic analyses.

**Methods:**

Interviews were undertaken with 100 member of the general population. Health state valuations were elicited using the time trade-off approach with a ten year time horizon. Additionally, respondents were asked where the EQ-5D response scale descriptors of moderate and extreme pain lay on the 11-point NPRS scale.

**Results:**

625 valuations were undertaken across the study sample with the crude mean health state utilities showing a negative non-linear relationship with respect to increasing pain intensity. Relative to a NPRS of zero (NPRS0), the successive pain levels (NPRS1-10) had mean decrements in utility of 0.034, 0.043, 0.061, 0.121, 0.144, 0.252, 0.404, 0.575, 0.771 and 0.793, respectively. When respondents were asked to mark on the NPRS scale the EQ-5D pain descriptors of moderate and extreme pain, the median responses were '4' and '8', respectively.

**Conclusions:**

These results demonstrate the potential floor effect of the EQ-5D with respect to pain and provide estimates of health reduction associated with pain intensity described by the NPRS. These estimates are in excess of the decrements produced by an application of the EQ-5D scoring tariff for both the United States and the United Kingdom.

## Background

The use of cost-effectiveness analysis has become an important part of the health technology assessment process [1]. Integral to this is the accurate measurement and valuation of quality of life. Whilst the problems associated with defining, describing and measuring health have been long known, additional problems are created when values capable of being incorporated into cost-effectiveness analysis are derived. These values, referred to as 'utilities', require specific properties, most notable of which is that they are anchored on two values; one and zero, representing full health and death (or a health state considered to be equally preferable to death). Only with this property can the utility values be multiplied against length of life to produce quality adjusted life years (QALYs). Intended to be a generic measure of health effects, QALYs allow a fuller assessment of cost-effectiveness through comparability across health care programs [2].

Health state utilities are produced in a number of different ways, but the most common is the use of generic preference based measures (PBMs). PBMs are a specific type of patient reported outcome measure; so questionnaires such as the EQ-5D are completed by patients and then a pre-existing tariff is applied to generate utility values [2]. However, the relevance of PBMs to all conditions has been called into question with evidence of poor measurement properties for some patient populations, including insensitivity to change and floor effects [3]. Floor effects exist when the lowest values of ill health or functioning are not represented by a patient reported outcome measure. As such, some respondents would actually describe their health or functioning as worse that the lowest category. This has two effects; firstly, the score for these respondents is biased upwards (on a scale where higher scores represent better health or functioning) and secondly, any change in health or functioning for these respondents is underestimated, thereby contributing to insensitivity to change.

Pain is a domain in all the main generic PBM descriptive systems, including the EQ-5D [4], SF-6D [5] and HUI-III [6]. However, there are concerns with the measurement properties of these instruments with respect to pain [7-10]. In purely descriptive validity terms, the EQ-5D is particularly open to criticism with only three levels of pain; none, moderate and extreme. The SF-6D and HUI-III offer greater sensitivity to changes due to the use of 6 and 5 levels, respectively. However, it unclear whether better descriptions are offered for their most severe levels. The severest level of pain as described within the SF-6D is, "You have pain that interferes with your normal work (both outside the home and housework) extremely" and the description within the HUI-III is "Severe pain that prevents most activities". It should be noted that the SF-6D descriptive system that forms the basis of its scoring algorithm is derived from that of the SF-36 [11] and is formed by combining both of the pain items from the SF-36 into a single domain. As such, the SF-6D descriptive system, is a simplification of the underlying SF-36.

A systematic review and meta-analysis of utilities in patients with neuropathic pain has been undertaken which showed that utilities varied across conditions, and was correlated with pain intensity as measured by the NPRS [8]. However, analyses were not provided that examined potential floor effects or sensitivity to change relating to any of the PBMs.

Whilst PBMs may have problems describing the full range of pain intensity, several clinical measures do not suffer from this problem. Studies evaluating the measurement properties of the NPRS, for example, show that it is sensitive to changes in pain intensity with high response rates [12]. From this we conclude that the measurement range of the NPRS is valuable in describing even the most severe levels of pain, and the number of levels makes it sensitive to clinically relevant changes in pain.

In this study we attempt to address the perceived floor effects and lack of sensitivity of the pain dimension of the EQ-5D by replacing its three point scale with the eleven point NPRS. The objectives of the study are to value a series of health states that incorporate the NPRS as a description of pain intensity and to calculate decrements in health utility associated with increasing severity of pain.

## Methods

### Interview schedule

An interview schedule was constructed that consisted of 5 sections. In the first, the respondent was asked to complete the EQ-5D to help them become accustomed to the idea of describing health in short statements using the EQ-5D descriptive system. In the second, four health states that replaced the EQ-5D pain dimension with the NPRS scale were presented and the respondent asked to rank the four health states from one to four, with '1' meaning the best health state and '4' the worst health state. In section three, a series of ten valuation tasks using a time trade-off (TTO) approach was presented (see 'TTO tasks'). Section four examined the relationship between the EQ-5D description of pain levels with the NPRS descriptive approach. In the first question the respondent was asked to mark on the NPRS where they felt 'moderate pain or discomfort' fell. In the second question the respondent was asked to mark on the NPRS where they felt 'extreme pain or discomfort' fell. Section five consisted of sociodemographic questions.

### TTO tasks

The TTO approach is used to produce utility values by asking resondents to identify a length of time (x) in full health that is equivalent to a longer duration (t) in a particular health state that is less than full health. The more an individual is willing to give up length of life in the health state, in exchange for full health, the less that health state is valued. The value x/t is the utility [13].

The duration of the health states was set at 10 years for all valuation tasks which is in line with the methods that underpin the EQ-5D valuation tariff [4]. Ten years in the selected health state was compared to varying durations of full health in tabular format on the questionnaire. The first line of the table stated that 'the [chosen] health state for 10 years followed by death is better than 0 years in full health followed by death' after which the respondent would place a tick, a cross or a question mark, depending on whether they agreed, disagreed or were uncertain, respectively. Subsequent lines increased the time in full health in increments of half a year, until the final line which stated that 'the (chosen) health state for 10 years followed by death is better than 10 years in full health followed by death', followed by the respondent's assessment.

In terms of Torrance's notation, the 10 years is t, the amount of time varied is x. The precise value of x used to calculate the utility of the selected health state was the mid-point between the values in the two statements where the 'cross' and 'tick' were closest together. In other words, when the respondent switched from agreeing to disagreeing with the statements.

In line with Torrance [13], if respondents considered the health state to be worse than death, which was indicated by a cross in the first row of the table described above, a further valuation task was undertaken to derive the necessary data to produce a health state value. This requires a more complex trade-off and different calculation to arrive at the utility, but in essence, it was formatted in the same way as before. A sequence of full health followed by the selected health state was compared to immediate death. The length of time in full health (x) plus the length of time in selected health state summed to ten years (t), with the length of time in the two component parts varied until it was considered of equal value to immediate death.

**Figure 1:**
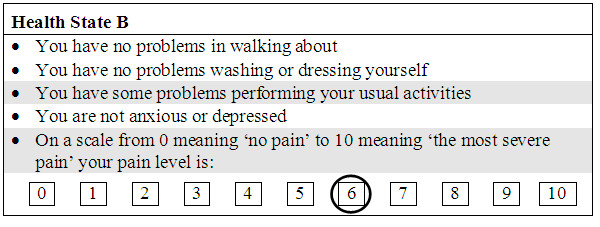
**Example of one of the health states used within the survey**.

The valuation tasks examined 11 health states with each containing one level of the NPRS, plus a further 7 health states that also included a further dimension describing other symptoms relating to common side-effects of medications. These additional 7 valuations are not used in the results presented in this paper and so are not described any further. A single EQ-5D health state was used as the basis for the NPRS valuations; no problems with mobility or self-care, some problems associated with usual activities but with no anxiety/depression (which can be abbreviated to '1121' using the convention of summarising the levels as numbers ranging from 1 to 3). An example of one of the health states valued is given in Figure 1.

The purpose of the valuation exercise was to produce utility decrements for the different levels of pain, and therefore, values were required for "no pain" plus the 10 pain levels of the NPRS (there are henceforth referred to as "nprs0" through to "nprs10"). When combined with the seven symptom states mentioned earlier, this required 18 health state valuation tasks, which was considered too cognitively demanding for respondents. Consequently, two interview schedules (marked 'A' and 'B') were constructed that were identical in structure and formatting, but differed only in the health states presented. One health state was replicated in both interviews to allow a test of consistency.

### Sample and interviewing

100 interviews with members of the general public were planned. The participants were approached in their own home, with houses (identified by their number and street) sampled at random from a list of addresses within three postal districts of the city of Cardiff. The postal districts were selected to reflect a range of sociodemographic characteristics, although no formal selection process was used for this.

All interviews were undertaken by a single trained interviewer. The precise formatting of the interview schedule was arrived at through a pilot study of seventeen members of the public. This also allowed the interviewer to familiarise themselves with the structure and routing of the interview schedule.

### Analysis

Health state values were calculated using the approach of Torrance [13]. For health states considered better than being dead, the time in full health considered to be equivalent to ten years ('t') in the target health state ('x') was divided by ten, i.e. utility = x/10. For health states considered to be worse than dead, the utility value is calculated as x/(x-t). All values were included in the analysis.

In the first of the analyses, means and incremental differences in means were described for each of the eleven NPRS levels. However, this ignores possible differences in values attributable to the different samples that received the two alternative interview packs. A multivariate analysis is therefore required to adjust for these differences, however, account also needs to be taken of the correlation between responses from the same individual. Therefore, coefficients were estimated using generalised estimating equations with robust standard errors and an exchangeable autocorrelation matrix in STATA v9.

Additionally, checks of validity and consistency that had been built into the study design were undertaken. The first of these compared the rankings within Section two and the TTO values generated from the responses in Section three. Convergent validity would be shown if the direct ranking matched the implied ranking using the derived TTO values. The second test compared the values of the health state that was valued in both versions of the interview schedule. No statistically significant differences between the values would suggest that the different contents of the schedules did not influence responses unduly.

Finally, the NPRS ratings of the EQ-5D pain descriptors were calculated. This would give an indication of the extent to which the descriptors covered the range of pain represented by the NPRS.

## Results

**Figure 2:**
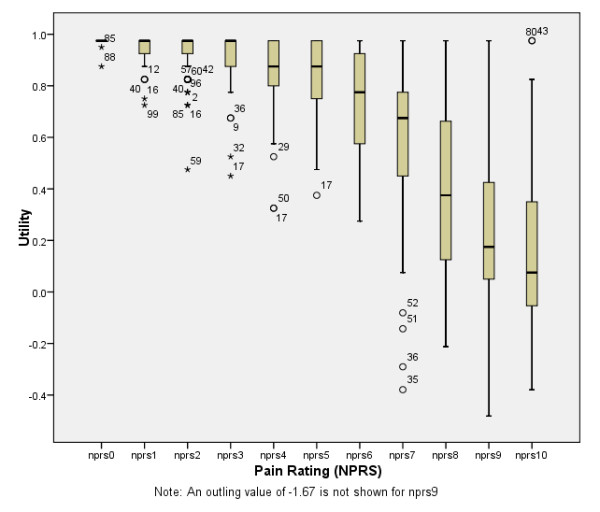
**Crude values and distributions for health states**.

Some differences were apparent between the sample interviewed with the two packs, with slightly more men and people with lower levels of formal education being interviewed with pack B (Table 1). When the crude utilities are calculated for all NPRS levels, a monotonically decreasing relationship is seen (Table 2). The relationship between utility and pain intensity appears to be non-linear and the distribution of values skew toward lower values except for NPRS levels 8, 9, 10 which appear approximately normally distributed (Figure 2).

**Table 1:** Sociodemographic characteristics of the sample split by survey

Characteristic	Survey A	Survey B
		

**Number of respondents**	48	52

		

**Age (SD)**	40.5 (16.1)	41.8 (15.3)

		

**Gender (% female)**	58.3	50.0

		

**Highest qualification**		

**GCSE or equivalent**	31.8	39.6

**HND/BTEC or equivalent**	6.8	8.3

**A level or equivalent**	22.7	10.4

**Degree or PhD**	38.6	41.7

		

**Occupation**		

**Professional**	27.1	30.8

**Managerial or technical**	20.8	19.2

**Manual skilled**	16.7	13.5

**Non-manual skilled**	8.3	15.4

**Partly skilled**	18.8	17.3

**Unskilled**	4.2	1.9

**Never had a job**	4.2	1.9

**Table 2:** Crude means for different NPRS health states

Health state	N*	Minimum	Maximum	Mean	Std. Deviation	Deviation from full health	Deviation from nprs0
**nprs0**	73	0.875	0.975	0.973	0.012	0.027	

**nprs1**	48	0.725	0.975	0.939	0.065	0.061	0.034

**nprs2**	100	0.475	0.975	0.931	0.085	0.069	0.043

**nprs3**	52	0.45	0.975	0.912	0.115	0.088	0.061

**nprs4**	52	0.325	0.975	0.852	0.153	0.148	0.121

**nprs5**	52	0.375	0.975	0.829	0.157	0.171	0.144

**nprs6**	48	0.275	0.975	0.721	0.217	0.279	0.252

**nprs7**	52	-0.379	0.975	0.569	0.319	0.431	0.404

**nprs8**	48	-0.379	0.975	0.398	0.349	0.602	0.575

**nprs9**	48	-1.667	0.975	0.202	0.449	0.798	0.771

**nprs10**	52	-0.379	0.975	0.180	0.327	0.820	0.793

* Pack A had 48 respondents, and pack B had 52 respondents. NPRS2 was in both packs. NPRS 0 was missing from both packs but added part way through the project to both packs.

For the multivariate analysis, 625 observations were available, with the mean number of observations per respondent being 6.3. The intraclass correlation was 0.033 (95% confidence interval, 0.000 to 0.089). The coefficients for the decrements in utility from full health (i.e. one) are consistent with the crude means, with only two respondent characteristics - interview length and job type - having a statistically significant influence on responses (Table 3). Only nprs6 through to nprs10 have statistically significant coefficients. The 95% confidence intervals for nprs9 and nprs10 incorporated health state values of less than zero.

**Table 3:** Decrements from full health adjusted for correlations and respondent characteristics

Independent variables	Coefficient (decrements from full health)	95% confidence interval of coefficient
**nprs0**	0.030	(-0.180 - 0.240)

**nprs1**	0.066	(-0.140 - 0.272)

**nprs2**	0.073	(-0.133 - 0.279)

**nprs3**	0.090	(-0.123 - 0.304)

**nprs4**	0.150	(-0.065 - 0.365)

**nprs5**	0.174	(-0.042 - 0.389)

**nprs6**	0.283	(0.077 - 0.490)**

**nprs7**	0.434	(0.207 - 0.660)**

**nprs8**	0.607	(0.397 - 0.817)**

**nprs9**	0.803	(0.598 - 1.008)**

**nprs10**	0.822	(0.602 - 1.043)**

		

**Own nprs level**	-0.013	(-0.028 - 0.002)

**gender**	0.023	(-0.048 - 0.094)

**age**	-0.001	(-0.002 - 0.001)

**ed2-4^+^**	-	

**job2-7^++^**	-	*

**Self-assessed difficulty**	-0.022	(-0.056 - 0.011)

**Length of interview**	0.005	(0.002 - 0.007)**

Key

* significant at 5%

** significant at 1%

^+ ^four education levels were possible. These have been presented as a single variable with the significance tested on all coefficients being zero.

^++ ^seven job types were possible. These have been presented as a single variable with the significance tested on all coefficients being zero.

A test of the trend in utility values in relation to the NPRS levels was undertaken by fitting curves to the estimated mean values from the multivariate analysis described above. A quadratic curve, estimated as *U = 0.957 +0.015 NPRS - 0.10 NPRS^2^*, was found to fit the data very well with an R-squared of 0.980 and a p-value of less than 0.001.

When respondents were asked to mark on the NPRS scale the EQ-5D pain descriptors of moderate and extreme pain, the median responses were '4' and '8', respectively (Table 4). A comparison of values for nprs2 from each of the two interview packs, using an independent samples t-test, showed a statistically significant difference of 0.061 (p < 0.001). This indicates that either the sample characteristics impacted on the values, or the ordering of the health state value had an effect. An ordering effect is possible as nprs2 health state was positioned fourth and 1^st ^in the A and B packs, respectively. A comparison of nprs0, which was added to both packs part way through the interviews (n = 73), showed no statistically significant difference in values (p = 0.486). An ordering effect is not possible with this comparison as the nprs0 health state was the final question in both Pack A and Pack B.

**Table 4:** Comparison of EQ-5D and NPRS pain levels

EQ-5D level	NPRS level (n = 100)	
	Mean (SD)	Median (25^th ^centile, 75^th ^centile)

**Moderate pain**	3.76 (1.138)	4.00 (3.00, 5.00)

**Extreme pain**	8.13 (1.012)	8.00 (8.00, 9.00)

A validity check between rankings (Section two) and valuations (Section three) was possible for Pack A for the nprs2 and nprs6 health states. Other checks within Pack A and all checks within Pack B involved health states with an additional symptom domain and so is outside the remit of this paper. For 34 of the 48 respondents, the ranking was consistent with the TTO valuation (i.e. nprs2 was ranked better than nprs6, and the TTO valuation of nprs2 was higher than that for nprs6). For 5 out of 48, nprs2 was ranked lower than nprs6, and for 9 out of 48, the TTO value for nprs2 and nprs6 was the same.

Overall 37% of the sample rated the difficulty of the valuation exercises as 'difficult' or 'very difficult'. Only 6% rated them as 'very difficult'.

## Discussion

This study used a novel approach to elicit utility values associated with different intensities of pain as measured by the NPRS. The approach adopted involved replacing the three point verbal pain scale that is integral to the EQ-5D, with the 11-point NPRS, which is recommended for clinical research of chronic pain [14]. A series of health states were then constructed around a fixed state defined in terms of mobility, self-care, usual activities and anxiety/depression, but with pain intensity varying from zero ('no pain') to 10 ('worst imaginable pain'). This approach was adopted in an attempt to use a validated descriptive system, but enhance its sensitivity and range of measurement with respect to pain.

The valuations were completed by all participants, albeit, with a small number of responses that were counterintuitive. The sample mean utilities were monotonically decreasing with respect to pain intensity, with increasing utility decrements as pain intensity increased. The multivariate analysis showed a very similar pattern with respect to utility decrements and showed that those decrements for nprs6 through to nprs10 were statistically significantly different from zero.

The results allow for a much greater range of pain to be valued in economic evaluations of interventions relating to pain management. 50% of respondents considered the most intense level of pain on the EQ-5D to be either NPRS8 or lower, which reinforces previous findings of floor effects with respect to the pain dimension of the EQ-5D. Likewise, the maximum decrement relating to pain using the United Kingdom tariff [15] is 0.269 (or 0.655 if the n3 term is also attributed solely to extreme pain)] and 0.537 for the United States tariff [16] (excluding any D1, I3 or I3-squared effects), compared to 0.822 in this valuation study. These differences suggest that the EQ-5D underestimates the benefits of the treatment of higher pain intensities, and as such, the associated economic evaluations potentially underestimate the cost-effectiveness of these pain management interventions.

Despite the innovative approach, there are weakness to the study. The first problem to consider is the use of a single health state on which to add the NPRS. This design feature was used so that simple, additive decrements related to the intensity of pain could be easily constructed. At this moment in time, we do not know to what extent the results are generalisable to other health states.

A second problem is the design of the health states that were presented to the respondents. Whilst the presentation of EQ-5D descriptors is straightforward within valuation studies, with the format for each dimension being the same, the NPRS is a marked deviation from this (Figure 1). The added prominence of the scale lent to it by being different, may have caused respondents to give additional weight to this dimension of health. This may have been exaggerated further by moving the NPRS to the end of the health state, whereas if it had been a straight replacement for the EQ-5D pain dimension, it would have been fourth. The need for this formatting change, however, was strongly indicated in the piloting work as several respondents found the switching between narrative and numeric scaling to be distracting. A further deviation from the EQ-5D descriptive system is that the NPRS refers only to pain, whilst the dimension that it replaced refers to 'pain or discomfort'.

Whilst we are unable to test whether the prominence of the NPRS could have contributed to greater weight being given to pain ratings, we can compare the mean utility value for the NPRS0 health state and the corresponding EQ-5D health state tariff value (11211). This is perhaps a narrower test of the impact of formatting differences on responses as any added prominence of 'no pain' should have no effect. This shows the EQ-5D tariff value to be 0.883 compared to the estimated value from our multivariate analysis of 0.970, which indicates a possible impact of the design on utility values. However, differences between the sample, and the format of the elicitation techniques would also be expected to contribute to differences in responses.

Most studies that have examined utilities in patient populations with pain have typically used PBMs [8]. McDermott [17], for example, reported EQ-5D values in 602 patients with neuropathic pain. Using the Brief Pain Inventory (BPI) Pain Severity score (which ranges from 0-10) to categorise pain as either 'mild' (1-3), moderate (4-6) or severe (7-10), Mc Dermott and colleagues calculated mean utilities of 0.67, 0.46 and 0.16, respectively.

Comparing these utilities to those in this study is difficult, because, although the BPI Pain Severity score has the same numerical scoring, the descriptor for point 10 on the scale is different to that for the NPRS, and additionally, the score used by McDermott was an average of four estimates; current pain, worst pain in the past 24 hours, least pain in the past 24 and average pain in the past 24 hours. However, the 'equivalent' mean utilities assuming an equal weighting for each level for NPRS1-3, NPRS 4-6 and NPRS7-10 are 0.93, 0.80 and 0.34. Even with the differences in the scales, and potential differences in the weighting for each level, these are quite stark discrepancies.

We expect that this is due to the patients within the McDermott study experiencing other pain-related impacts on their health, for example, their sample had higher rates of depression/anxiety and reduced working time. As such, our utility decrements associated with pain tend to underestimate the overall effect of pain on health related quality of life. How these additional effects can be combined with our NPRS based utility values is discussed later in this article.

Eldabe et al [18] took a different approach to estimating utilities for health states relating to severe chronic pain. Their approach was to develop bespoke health states describing intensity of pain in narrative format, together with other health impacts that were considered to be associated with the particular intensity of pain described. Each narrative description was supposed to indicate a different range of pain intensity as measured by the VAS-PI, so for example, VAS-PI 61-80 was described as "moderately severe pain that is hard to tolerate even with treatment". These pairings were devised through clinician interviews and piloting. Four levels of pain were described and valued using a TTO approach with health states having a duration of 5 years.

Comparisons with our study are again difficult, but suggest decrements compared to VAS-PI 0-40 of 0.12, 0.69 and 1.03 for VAS-PI 41-60, VAS-PI 61-80 and VAS-PI 81-100, respectively. These much greater differences to the results presented here are again thought to be primarily due to the co-morbid effect of pain on other aspects of daily life. These decrements are also noticeably greater than those reported by McDermott.

The simplest approach to using the NPRS utility decrements described in this paper is to apply them to NPRS data within trials to calculate a utility difference between a control and intervention group. However, as noted previously, this does not take into account the co-morbid effects of pain on other aspects of health related quality of life. A direct consequence of this is that the utility gain of reductions in pain may be underestimated.

Therefore, the NPRS decrements should be used in tandem with EQ-5D data collected from patients within the clinical trials. For any set of EQ-5D from a questionnaire, the EQ-5D scoring algorithm can be applied to the four non-pain dimensions, then the decrement with respect to their NPRS should then be applied. In this way, any improvement in mobility, self-care, usual activities and depression/anxiety related to improvements in pain control would also be captured.

In terms of pain utility values, our approach needs further work. Firstly, an examination of the effect that formatting has on responses needs to be undertaken as the possibility of a 'prominence effect' may lead to biases in the utility values produced. Secondly, exploratory work needs to be undertaken to see the extent to which the NPRS may precipitate other alterations to the EQ-5D tariff. Only if pain, as measured by the NPRS remains independent of the other domains, and does not affect their weighting, can the NPRS utility decrements be legitimately combined with EQ-5D tariff based scores in the way suggested above. The easiest way to examine this is to undertake valuation studies of a selection of EQ-5D health states and analogous 'EQ-5D-NPRS' health states within the same study sample, then test for differences in the values produced. A more complex approach would be to re-estimate a completely new tariff for the 'EQ-5D-NPRS' and test for differences with the existing EQ-5D tariff (or a new tariff based on a new valuation study).

The approach reported here was found to produce a set of values that had face validity - non-linear relationship with respect to pain intensity - and which had a high level of internal consistency among respondents. However, the valuations produced in this paper are limited by their exclusion of the co-morbid effects of pain on other dimensions. As such, they need to be combined with PBM data in order to fully estimate the health related quality of life impacts of pain. In order to assess the validity of this 'mix and match' approach, further research is needed to assess the independence of other scales when incorporated within health states based on the EQ-5D using the approaches highlighted above

## Conclusions

These results demonstrate the floor effect of the EQ-5D with respect to pain and provide estimates of health reduction associated with pain intensity described by the NPRS. These estimates are in excess of the decrements produced by an application of the EQ-5D scoring tariff for both the United States and the United Kingdom. However, their use in technology assessment is not straightforward as they do not capture the co-morbid effects of pain. Consequently, our estimates would have to be used in tandem with existing scoring algorithms to capture the full health effects of pain. Combining two validated measures in this way represents a valuable way of linking clinical and economic outcome measures, but further work is required in order to produce more robust utility estimates that can be used in technology assessment.

## List of abbreviations

NPRS: Numerical pain rating scale; PBM: Preference based measure; QALY: Quality adjusted life year; TTO: Time trade-off; VAS-PI: Visual analogue scale for pain intensity

## Competing interests

The study was funded by Astellas Pharma Ltd. Isaac Odeyemi, Peny Retsa and Colette Chambers are currently an employee of Astellas Pharma Ltd. Astellas manufacture products for pain relief.

## Authors' contributions

SD led the design and analysis of the project and drafting of the manuscript. CP, CJC, IO, PS and CC contributed to the design and interpretation of the project and the drafting of the manuscript. All authors have read and approved the manuscript.
